# Severely displaced rib fractures are independently associated with reduced pulmonary function at 3 months

**DOI:** 10.1186/s13017-025-00667-7

**Published:** 2025-12-11

**Authors:** Yu-Hao Wang, Szu-An Chen, Yu-San Tee, Ling-Wei Kuo, Chi-Tung Cheng, Sheng-Yu Chan, Shih-Ching Kang, Chi-Hsun Hsieh, Fausto Catena, Chien-An Liao

**Affiliations:** 1 Department of Trauma and Emergency Surgery, Linkou Medical Center, Chang Gung Memorial Hospital, No. 5, Fuxing St., Guishan Dist., Taoyuan City, 33305 Taiwan; 2 Department of Medical and Surgical Sciences, University of Bologna, Bologna, Italy; 3 Department of General and Emergency Surgery, Bufalini Hospital, Cesena, Italy; 4 Department of Biomedical Engineering, National Taiwan University, Taipei, Taiwan; 5 Chang Gung University College of Medicine, Taoyuan, Taiwan

**Keywords:** Rib fractures, Blunt chest trauma, Displaced rib fractures, Pulmonary ventilation function

## Abstract

**Introduction:**

Multiple rib fractures are common injuries resulting from blunt chest trauma. However, the effect of rib fracture displacement on pulmonary ventilation remains unclear. This study aimed to investigate the effect of severely displaced ribs on pulmonary ventilation function (PVF) 3 months post-trauma.

**Materials and methods:**

This retrospective case–control study was conducted at Chang Gung Memorial Hospital. Patients with multiple rib fractures (≥ 3) who underwent chest computed tomography (CT) from January 2019 to September 2023 were included. Patient demographics, injury severity, and rib fracture morphology were assessed. Displaced rib fractures were defined as bicortical displacements observed on CT. PVF was measured using forced vital capacity (FVC) and forced expiratory volume in 1 s. Univariate and multivariate logistic and linear regression analyses were performed to determine whether displaced rib fractures significantly affected PVF 3 months post-trauma.

**Results:**

Overall, 111 patients with multiple rib fractures were included. Displaced rib fractures were identified as an independent risk factor for having FVC < 80% at 3 months post-trauma, with each additional severely displaced rib increasing the odds by 31% (odds ratio: 1.31, 95% CI 1.09–1.57, *p* = 0.004). Subgroup analysis revealed that this effect was particularly significant in patients with non-flail chests. The receiver operating characteristic curve and Youden index identified that the optimal cutoff value for significantly displaced rib fractures affecting PVF was three or more fractures.

**Conclusion:**

Severely displaced rib fractures significantly impact PVF 3 months post-trauma.

**Supplementary Information:**

The online version contains supplementary material available at 10.1186/s13017-025-00667-7.

## Introduction

Multiple rib fractures are the most common injuries resulting from blunt chest trauma, with incidence rates of 39–58% in patients with blunt chest trauma and 5–10% in those with all types of trauma [1–4]. Rib cage deformity decreases pulmonary ventilation function (PVF), increasing pulmonary complications and mortality rates, especially in patients with a flail chest—a chest wall deformity caused by fractures in three consecutive segmental ribs [5–9].

Surgical rib fixation has significantly increased over the years [10, 11]. Currently, the most robust evidence shows that surgical rib fixation is favorable for patients with flail chest. In these patients, surgical intervention reduces pain, decreases pulmonary complications, and shortens the duration of mechanical ventilator use [12]. Additionally, rib fixation in flail chest improved PVC within 3 months [13]. However, the indications for rib fixation in patients with a non-flail chest remain unclear. A prospective study suggested that severely displaced rib fractures are an indication of a non-flail chest [14]. Displacement severity is related pain intensity [15]. Therefore, although rib fixation may benefit these patients, supporting evidence is scarce. Furthermore, the effect of severely displaced rib fractures on respiration remains unclear. Therefore, we aimed to determine the impact of the number of severely displaced ribs on PVF at the 3-month follow-up.

## Materials and methods

### Study design and patient selection

This single-center, retrospective, case–control analysis was conducted at Chang Gung Memorial Hospital. The selected cohort comprised adult patients (age > 16 years) with multiple rib fractures (≥ 3) who underwent chest CT scans at our institution between January 2019 to September 2023. Patients with severe head trauma (head abbreviated injury scale (AIS) score ≥ 4), in-hospital mortality, or missing 3-month PVF data were excluded.

The collected data included demographic details, medical history (including chronic obstructive pulmonary disease (COPD) or asthma), other comorbidities, smoking history, trauma mechanism, injury severity (measured using the AIS), associated injuries. We defined lung contusion as a score of at least 4 points on the Blunt Pulmonary Contusion 18 (BPC18) scoring system, which includes the moderate (4–6 points) and severe (7–18 points) categories. Surgical rib fixation details (including interval and number of ribs fixed), severe hemothorax (defined as > 50% lung cavity in either plain film or CT, and pneumothorax were also recorded. Rib fracture score was calculated as the total number of rib fractures, multiplied by 2 if there are bilateral fractures, plus an age factor (51–60 = 1; 61–70 = 2; 71–80 = 3; > 80 = 4) to reflect general disease severity [16].

### Morphology of rib fracture

The morphology of the rib fractures was assessed using chest CT scans, categorizing them as unilateral or bilateral. Flail chest was defined as the “radiographic flail,” which is the presence of three or more adjacent ribs fractured in two or more places, as confirmed by chest computed tomography (CT) scans. Fracture sites were classified as lateral, posterior, or combined. Severely displaced rib fractures were defined as exhibiting bicortical displacement, displacement, or ≥ 100% displacement.

### Indication of rib fixation in our facility

In this cohort, surgical rib fixation was performed based on three specific indications:

1. Flail Chest: This includes patients with a clinically evident flail chest or a radiologically confirmed flail chest.
2. Severely Displaced Rib Fractures: This includes cases with severely displaced rib fractures, typically defined as having a displacement of more than one cortex.
3. Other Associated Conditions: For patients without a flail chest or severely displaced fractures, surgery was indicated for impending or actual respiratory failure not attributable to lung contusion, inability to wean off of mechanical ventilation, or inadequate pain control.

### Follow-up protocol and pulmonary function test

Patients underwent pulmonary function tests 3 months post-trauma. We employed a predictive equation to calculate the expected lung function based on patient age and height[17]. The ratios of the measured to predicted values were used as observational indices for subsequent statistical analyses. PVF measurements included Forced vital capacity (FVC) and forced expiratory volume in 1 s (FEV_1_). All measurements were routinely performed by a respiratory specialist, using a portable pulmonary function detector. Each index was measured three times, and the mean values were used for the ratio calculations.

### Statistical analysis

SPSS 29.0 (SPSS Inc., Chicago, IL, USA) was used for statistical analysis. Descriptive statistics are expressed as numbers and percentages for categorical variables and as means, standard deviations, minima, and maxima for numerical variables. For comparisons between the two groups, an independent t-test was used for numerical variables, Pearson’s chi-square test for large-sample-sized categorical variables, and Fisher’s exact test for small-sample-sized categorical variables. Univariate and multivariate logistic regression analyses were conducted, with “low forced vital capacity (< 80%)” used as the dependent variable to determine the risk factors for deteriorating pulmonary function. The receiver operating characteristic (ROC) curve and Youden index were used to determine the best cutoff value for the number of displaced rib fractures affecting pulmonary vital capacity. A *p* value < 0.05 was considered significant.

## Results

**Fig. 1:**
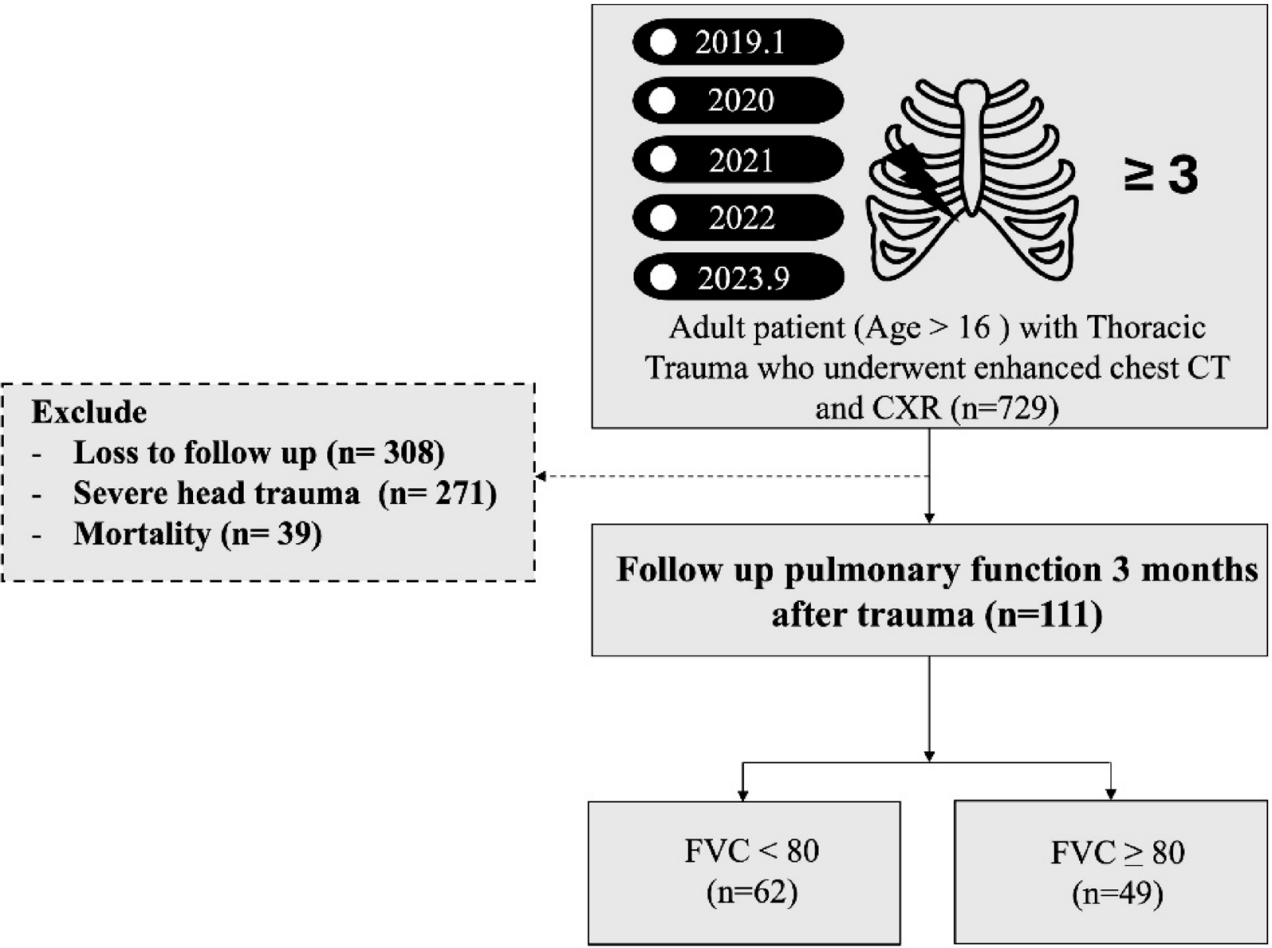
Flowchart of the patient selection protocol of the present study. This flow diagram illustrates the study process and outcomes in a cohort of patients with thoracic trauma who underwent enhanced chest CT and CXR between January 2019 and September 2023. The number of patients initially evaluated is 729. Patients lost to follow-up (n = 308), those with severe head trauma (n = 271), or those who died (n = 39) were excluded. The remaining 111 patients underwent follow-up pulmonary function testing 3 months post-trauma. These patients were categorized based on their forced vital capacity (FVC) values: 62 patients had FVC < 80, and 49 patients had FVC ≥ 80

Overall, 729 eligible patients were identified. Figure 1 shows the patient selection flowchart**.** Ultimately, 111 patients were included; their key demographic and clinical details are reported in Table 1. Among the 73 patients who underwent rib fixation, 67 (91.7%) had severely displaced rib fractures, and 38 (52.1%) had a flail chest. Only six (8.2%) patients underwent rib fixation for other reasons, such as respiratory failure or uncontrolled pain, without having either a flail chest or a severely displaced fracture. Patients were divided into two groups based on their FVC at the 3-month follow-up: the FVC ≥ 80% group (n = 49) and the FVC < 80% group (n = 62) (Table 2). Patients with an FVC of < 80% had significantly higher Rib Fracture score (mean 8.4 vs. 11.2, *p* value: 0.026) and more rib fractures than did those with an FVC of ≥ 80% (*p* = 0.033). The number of severely displaced rib fractures was also significantly higher in the FVC < 80% group than in the FVC ≥ 80% group (*p* = 0.003). Bilateral rib fractures were more common in the FVC < 80% group than in the FVC ≥ 80% group (*p* = 0.043). Flail chest was observed in 48.4% of patients with an FVC of < 80% and in 32.7% of patients with an FVC of ≥ 80%, although this difference was not significant (*p* = 0.095). No significant differences were observed in age, sex, smoking status, presence of COPD or asthma, associated injuries, injury severity score (ISS), or lung contusions between the two groups. While the rib fixation rate was similar between the two groups, time from injury to rib fixation was significantly longer in the FVC < 80% group (4.6 ± 2.2) compared with the FVC ≥ 80% group (3.8 ± 1.5) (*p* = 0.044).

**Table 1:** Characteristics of the study cohort (n = 111)

Variable	n (%), Mean ± SD (min–Max)
Sex (male/female)	75 (67.6%)/36 (32.4%)
Age	57.3 ± 14.2 (17 − 87)
Smoking	28 (25.2%)
COPD^a^ or asthma	3 (2.7%)
Mechanism	
Motorcycle collision	75 (67.6%)
Fall	22 (19.8%)
Motor vehicle collision	6 (5.4%)
Pedestrian	3 (2.7%)
Bicycle	3 (2.7%)
Other	2 (1.8%)
Associated injury	
Head AIS^b^ ≥ 3	9 (8.1%)
Chest AIS^b^	
4	105 (94.6%)
5	6 (5.4%)
Abdominal AIS^b^ ≥ 3	5 (4.5%)
ISS^c^	21.3 ± 5.4 (16–41)
Rib fracture score	9.95 ± 6.4 (4–44)
Lung contusion	88 (79.3%)
Pneumothorax	51 (45.9%)
Severe hemothorax^d^	6 (5.4%)
Rib fracture morphology	
Patients with bilateral ribs fracture	15 (13.5%)
Patients with flail chest	46 (41.4%)
Number of fractured ribs	7.1 ± 2.7 (3–22)
Patients with severely displaced fractures	93 (83.8%)
Number of severely displaced fractures	3.2 ± 2.5 (0–14)
Fracture site	
Lateral	19 (17.1%)
Posterior	16 (14.4%)
Combined	76 (68.5%)
Patients with rib fixation	73 (65.8%)
Number of ribs surgically fixed	3.9 ± 1.2 (2–7)
Length of hospital stay	11.2 ± 6.0 (2–36)
Length of ICU stay	2.7 ± 3.9 (0–18)
Pulmonary function test (3 months post-trauma)	
FVC^e^	77.2 ± 20.2 (40–125)
FEV_1_^f^	77.8 ± 19.7 (25–133)

^a^Chronic obstructive lung disease

^b^Abbreviated injury scale

^c^Injury severity score

^d^Hemothorax more than half of lung volume

^e^Forced vital capacity f. Forced expiratory volume in 1 s

**Table 2:** Comparison of patients with FVC^e^ ≥ 80% and FVC^e^ < 80% 3 months post trauma

	FVC^e^ ≥ 80% (n = 49)	FVC^e^ < 80% (n = 62)	*p* value
Sex (male/female)	32 (65.3%)/17 (34.7%)	43 (69.4%)/19 (30.6%)	0.687
Age	55.8 ± 14.2 (17–84)	58.5 ± 14.3 (26–87)	0.322
Smoking	11 (22.4%)	17 (27.4%)	0.661
COPD^a^ or asthma	0 (0.0%)	3 (4.8%)	0.254
Head AIS^b^ ≥ 3	6 (12.2%)	3 (4.8%)	0.180
Chest AIS^b^			0.692
4	47 (95.9%)	58 (93.5%)	
5	2 (4.1%)	4 (6.5%)	
Abdominal AIS^b^ ≥ 3	2 (4.1%)	3 (4.8%)	1.000
ISS^c^	21.0 ± 5.4 (16–36)	21.6 ± 5.4 (16–41)	0.597
Rib Fracture Score	8.4 ± 4.4 (4–26)	11.2 ± 7.5 (4–44)	0.026
Lung contusion	35 (71.4%)	53 (85.5%)	0.070
Pneumothorax	20 (40.8%)	31 (50.0%)	0.335
Severe hemothorax^d^	4 (8.2%)	2 (3.2%)	0.403
Rib fracture pattern			
Patients with bilateral ribs fracture	3 (6.1%)	12 (19.4%)	0.043
Patients with flail chest	16 (32.7%)	30 (48.4%)	0.095
Number of fractured ribs	6.5 ± 2.1 (4–13)	7.6 ± 3.0 (3–22)	0.033
Patients with severely displaced fractures	37 (75.5%)	56 (90.3%)	0.360
Number of severely displaced fractures	2.4 ± 2.2 (0–9)	3.8 ± 2.6 (0–14)	0.003
Fracture site			0.068
Lateral	12 (24.5%)	7 (11.3%)	
Posterior	9 (18.4%)	7 (11.3%)	
Combined	28 (57.1%)	48 (77.4%)	
Patients with rib fixation	44 (71.0%)	29 (59.2%)	0.194
Number of ribs surgically fixed	3.9 ± 1.2 (2–7)	3.9 ± 1.1 (2–6)	0.906

^a^Chronic obstructive lung disease

^b^Abbreviated injury scale

^c^Injury severity score

^d^Hemothorax more than half of lung volume

^e^Forced vital capacity

^f^Forced expiratory volume in 1 s

Univariate and multivariate logistic regression analyses were conducted to identify factors influencing lung function 3 months post-trauma **(**Table 3**)**. In the univariate analysis, several factors were associated with FVC of less than 80%. Flail chest and bilateral rib fracture demonstrated odds ratios (ORs) of 1.93 (95% confidence interval (CI): 0.89–4.21, *p* = 0.096) and 3.68 (95% CI 0.98–13.87, *p* = 0.054), indicating a significant difference. The number of rib fractures and fracture sites classified as combined were significantly associated with lower FVC (OR: 1.20, 95% CI 1.01–1.43, *p* = 0.039 and OR: 2.57, 95% CI 1.13–5.85, *p* = 0.024, respectively). Notably, severe displacement showed a significant impact (OR: 3.03, 95% CI 1.04–8.78, *p* = 0.041). The number of severely displaced fractured ribs also differed significantly between the groups. Each additional severely displaced rib fracture increased the odds of a patient having FVC < 80% at 3 months post-trauma by 31% (OR: 1.31, 95% CI 1.09–1.57, *p* = 0.004).

**Table 3:** Logistic regression analysis of factors affecting lung function (FVC < 80%) 3 months post-trauma

	Univariate logistic regression		Multivariate logistic regression	
	Odds Ratio (95% CI)	*p* value	Odds Ratio (95% CI)	*p* value
Sex (male/ female)	1.20 (0.54 to 2.67)	0.651		
Age	1.01 (1.00 to 1.04)	0.319		
Head AIS^a^	0.74 (0.47 to 1.17)	0.193		
Chest AIS^a^	1.62 (0.28 to 9.24)	0.587		
Abdominal AIS^a^	1.11 (0.72 to 1.71)	0.649		
Smoking	1.31 (0.55 to 3.12)	0.550		
Lung contusion	2.36 (0.92 to 6.03)	0.074	1.00 (0.30 to 3.30)	0.993
Pneumothorax	1.45 (0.68 to 3.09)	0.336		
Severe hemothorax^b^	0.38 (0.07 to 2.14)	0.269		
Patients with flail chest	1.93 (0.89 to 4.21)	0.096	0.73 (0.25 to 2.19)	0.577
Patients with bilateral ribs fracture	3.68 (0.98 to 13.87)	0.054	2.53 (0.48 to 13.27)	0.274
Number of fractured ribs	1.20 (1.01 to 1.43)	0.039	1.01 (0.78 to 1,30)	0.945
Fracture site				
Lateral	0.39 (0.14 to 1.09)	0.073		
Posterior	0.57 (0.19 to 1.65)	0.296		
Combined	2.57 (1.13 to 5.85)	0.024	0.73 (0.25 to 2.19)	0.297
Patients with severely displaced fractures	3.03 (1.04 to 8.78)	0.041		
Number of severely displaced fractures	1.31 (1.09 to 1.57)	0.004	1.27 (1.02 to 1.58)	0.032
Patients with rib fixation	1.69 (0.77 to 3.72)	0.195		
Number of ribs surgically fixed	0.98 (0.65 to 1.47)	0.904		

^a^Abbreviated injury scale

^b^Hemothorax more than half of lung volume

In the multivariate analysis, the number of severely displaced ribs remained a significant risk factor for decreasing PVF (adjusted OR: 1.27, 95% CI 1.02–1.58, *p* = 0.032). Other factors including sex; age; AIS scores for head, chest, and abdominal injuries; rib fixation; and smoking were not considered significant risk factors for decreasing PVF 3 months post trauma in the multivariate analysis.

The subgroup analysis revealed different factors affecting lung function in patients with and without flail chest (Table 4). Among patients without flail chest (n = 65), those with FVC < 80% had a significantly higher Rib Fracture Score (mean: 9.3 v.s. 6.8, *p* = 0.021), and higher incidence of bilateral rib fractures (15.6% vs. 0%, *p* = 0.024). They also had more rib fractures on average (6.6 ± 2.3 vs. 5.6 ± 1.4, *p* = 0.026) and more severely displaced fractures (2.9 ± 2.0 vs. 1.6 ± 1.6, *p* = 0.002). However, in patients with flail chest (n = 46), there were no significant differences between the FVC < 80% and FVC ≥ 80% groups in terms of Rib fracture Score, bilateral rib fractures, number of rib fractures, number of severely displaced fractures, rib fixation rates, or lung contusion rates. We further performed univariate and multivariate linear regression analyses in patients without flail chest and found that the number of severely displaced fractures severely affected pulmonary function 3 months post-trauma in both univariate and multivariate analyses (Table 5).

**Table 4:** Factors affecting lung function 3 months post-trauma in patients with and without flail chest

	FVC ≥ 80%	FVC < 80%	*p* value
Patient without flail chest (n = 65)	(n = 33)	(n = 32)	
Rib Fracture Score	6.8 ± 1.9 (4–12)	9.3 ± 5.5 (4–30)	0.021
Patients with bilateral ribs fracture	0 (0.0%)	5 (15.6%)	0.024
Fracture site			
Combined	12 (36.4%)	18 (56.3%)	0.108
Number of fractured ribs	5.6 ± 1.4 (4–8)	6.6 ± 2.3 (3–14)	0.026
Patients with severely displaced fractures	21 (63.6%)	27 (84.4%)	0.057
Number of severely displaced fractures	1.6 ± 1.6 (0–6)	2.9 ± 2.0 (0–6)	0.002
Patients with rib fixation	16 (48.5%)	19 (59.4%)	0.379
Lung contusion	20 (60.6%)	20 (62.5%)	0.126
Patient with flail chest (n = 46)	(n = 16)	(n = 30)	
Rib Fracture Score	11.6 ± 6.0 (6–26)	13.2 ± 8.8 (6–44)	0.532
Patients with bilateral ribs fracture	3 (18.8%)	7 (23.3%)	1.000
Fracture site			
Combined	16 (100.0%)	30 (100.0%)	1.000
Number of fractured ribs	8.2 ± 2.2 (4–13)	8.7 ± 3.4 (5–22)	0.305
Patients with severely displaced fractures	16 (100.0%)	29 (96.7%)	1.000
Number of severely displaced fractures	4.2 ± 2.2 (2–9)	4.8 ± 2.8 (0–14)	0.214
Patients with rib fixation	13 (81.3%)	25 (83.3%)	1.000
Lung contusion	15 (93.8%)	28 (93.3%)	1.000

**Table 5:** Subgroup analysis of factors affecting FVC (%) 3 months post-trauma in patients without flail chest (n = 65)

	Univariate linear regression	*p* value	Multivariate linear regression	*p* value
	B (95% CI)		B (95% CI)	
Patients with bilateral ribs fracture	− 17.18 (− 35.87 to 1.50)	0.071	− 5.62 (− 26.54 to 15.30)	0.593
Number of fractured ribs	− 2.73 (− 5.31 to − 0.15)	0.038	− 1.36 (− 4.36 to 1.63)	0.366
Fracture site: combined	− 10.21 (− 20.13 to − 0.28)	0.044	− 5.96 (− 16.88 to 4.97)	0.280
Number of severely displaced fractures	− 3.81 (− 6.36 to − 1.25)	0.004	− 3.03 (− 5.80 to − 0.25)	0.033
Patients with rib fixation	− 6.70 (− 16.81 to 3.42)	0.191		
Number of ribs surgically fixed	1.51 (− 6.99 to 10.01)	0.720		
Lung contusion	− 13.13 (− 23.69 to − 2.56)	0.016	− 2.37 (− 15.40 to 10.66)	0.717
Pneumothorax	− 0.95 (− 11.45 to 9.56)	0.858		
Severely hemothorax^a^	− 14.08 (− 55.45 to 27.29)	0.499		

^*^R-squared of the multivariate linear model 0.194; F: 2.792, Sig. 0.025

^*^FVC: Forced vital capacity

^a^Hemothorax more than half of the lung volume

Another subgroup analysis was performed based on whether patients underwent rib fixation. In patients who underwent rib fixation, we compared those with FVC ≥ 80% and FVC < 80%. The only significant difference was the number of severely displaced fractures (mean 3.21 vs. 4.52, *p* = 0.025), which suggests that even after rib fixation, a higher number of severe displaced fractures is associated with a poor pulmonary function outcome. Furthermore, in this subgroup, patients with multiple severe displaced fractures (≥ 3) had a poorer 3-month post-trauma FVC (mean: 72.4% vs. 83.5%, *p* = 0.038) and a higher proportion of patients with FVC < 80% (69.1% vs. 33.3%, *p* = 0.016). On the other hand, in patients without rib fixation, there was no significant difference in the number of severely displaced fractures between those with FVC ≥ 80% and FVC < 80%. Likewise, there was no significant difference in pulmonary function outcomes between the groups with and without multiple severe displaced fractures (≥ 3) (Supplementary Tables 1 and 2).

**Fig. 2:**
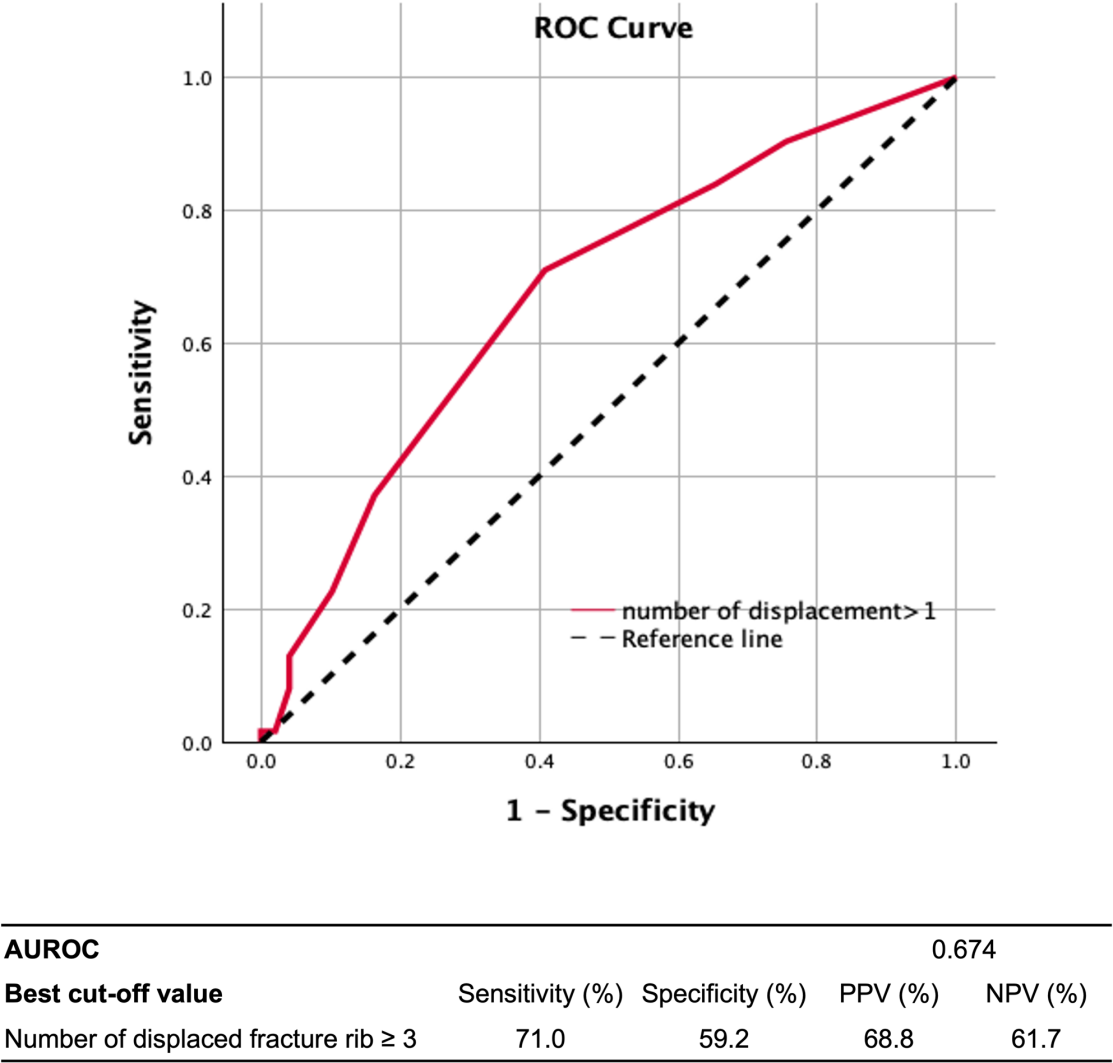
Number of severely displaced rib fractures and FVC < 80 at 3 months post-trauma—ROC curve analysis. The receiver operating characteristic (ROC) curve illustrates the diagnostic performance of the number of severely displaced rib fractures in predicting forced vital capacity (FVC) < 80 at 3 months post-trauma. The area under the ROC curve (AUROC) is 0.674, suggesting moderate discriminative ability. The best cutoff value identified for the number of displaced rib fractures is ≥ 3. At this threshold, the sensitivity is 71.0%, specificity is 59.2%, positive predictive value (PPV) is 68.8%, and negative predictive value (NPV) is 61.7%

To determine the optimal cutoff value for the number of severely displaced rib fractures affecting pulmonary function 3 months post-trauma, ROC curve analysis and Youden index were used. The analysis identified three or more severely displaced rib fractures as the optimal cutoff value with a sensitivity of 71.0%, specificity of 59.2%, positive predictive value of 68.8%, and negative predictive value of 61.7% (Fig. 2).

## Discussion

To our knowledge, this study is the first to investigate the correlation between displaced rib fractures and pulmonary function outcomes 3 months post-trauma. The definition of displaced rib fractures varies in the literature, with bicortical displacement being the most commonly cited [18]. Bugaev proposed a three-dimensional equation to evaluate the displacement; however, its complexity limited its practical application [19]. Previous studies have suggested that displaced rib fractures independently increase the risk of pneumonia and respiratory failure, and tracheostomy [15, 20–22]. However, no previous studies have specifically linked these fractures to long-term pulmonary function outcomes. Our study found that bicortical displacement in rib fractures significantly affected pulmonary function outcomes 3 months post-trauma.

Previous studies have investigated the correlation between displaced rib fractures and increased opioid use, which indicated more severe pain [15]. This pain may limit deep breathing capacity, leading to shallow breathing and atelectasis, which can further affect PVF. Another possible pathophysiological mechanism is that the increased displacement in rib fractures suggests higher impact energy, leading to increased tissue damage and inflammation. Although not previously demonstrated specifically in rib fractures, displaced fractures are a known risk factor for other bones’ nonunion [23–25]. These factors can predispose patients to a further compromised respiratory function.

Many medical facilities and clinical studies have considered significantly displaced rib fractures as indications for surgical rib fixation [26, 27]. Randomized multicenter trials have demonstrated that surgical fixation of non-flail chest rib fractures can significantly shorten hospital stay in intubated patients [28]. Pieracci et al. further observed a lower rate of intrapulmonary complications and reduced pain in patients with three or more displaced rib fractures who underwent surgical rib fixation [14]. However, our study did not find a significant difference in 3-month PVF outcomes between patients with and without surgical rib fixation. Selection bias occurred because patients with flail chest and severely displaced fractures underwent surgical rib fixation at our facility, indicating a higher severity of rib fractures in this group. Therefore, the efficacy of rib fixation for improving pulmonary ventilation remains unclear.

Subgroup analysis showed that, within the flail chest subgroup, no significant difference was found in displaced rib fractures between patients with and without pulmonary function impairment. This is largely due to the prevalence of displaced rib fractures in most patients with flail chest. However, a lower PVF tended to correlate with a higher number of displaced rib fractures, although this was not statistically significant. Conversely, in the non-flail chest subgroup, displaced rib fractures significantly affected PVF outcomes. This underscores the importance of displaced rib fractures as a crucial factor affecting PVF outcomes in patients with a non-flail chest, indirectly suggesting the potential benefits of surgical rib fixation in this subgroup.

When considering rib fixation, it's notable that even after surgery, patients with more severely displaced fractures had significantly worse pulmonary function. This highlights the importance of fracture severity. This may be due to more severe underlying damage and the fact that fixation often targets only the 3rd to 10th ribs, potentially missing other severe fractures. Conversely, in patients without rib fixation, there was no significant difference in pulmonary function. This could be due to a selection bias, as most patients with multiple severe fractures were likely referred for surgery. Additionally, while 60–70% of this group had displaced fractures, the average was only one or two, which may not be severe enough to significantly impact pulmonary function.

Our study had several limitations. First, the substantial loss to follow-up may have compromised the overall reliability of the results. Furthermore, the lack of long-term follow-up, such as at 6 months and 1 year, reduces the value of the findings. Second, we lacked pre-injury PVF data, which is a common challenge in trauma studies. Additionally, we lacked data on early postinjury pulmonary function, which would have provided a more comprehensive understanding of the immediate impact of rib fractures on pulmonary function. Furthermore, there was a lack of analysis regarding the quantity of hemothorax and pneumothorax, as well as the proportion of patients who used chest tubes. Third, we did not collect data on pain level or pulmonary complications, which are crucial factors in evaluating patient outcomes and treatment effectiveness. Finally, our study did not conclusively determine the efficacy of surgical rib fixation in the management of displaced rib fractures.

## Conclusions

Severely displaced rib fractures significantly impair PVF 3 months post-trauma, particularly in patients with a non-flail chest. Each additional severely displaced rib increased the odds of a patient having FVC < 80% at 3 months post-trauma by 31% (OR = 1.31), with three or more displaced ribs constituting the critical threshold. Therefore, more attention should be given to assessing rib displacement degree when managing chest trauma. Further research is required to evaluate the potential benefits of surgical rib fixation in patients with a non-flail chest and severe rib displacement.

## Supplementary Information

 Below is the link to the electronic supplementary material.

Supplementary Material 1

Supplementary Material 2

## Data Availability

The datasets generated and/or analyzed during the current study are available from the corresponding author upon reasonable request.

## Abbreviations

AIS: Abbreviated injury scale
CI: Confidence interval
COPD: Chronic obstructive pulmonary disease
CT: Computed tomography
CXR: Chest radiograph
FEV1: Forced expiratory volume in 1 s
FVC: Forced vital capacity
ISS: Injury severity score
OR: Odds ratio
PVF: Pulmonary ventilation function
ROC: Receiver operating characteristic
