# Supplementary material for: Severely displaced rib fractures are independently associated with reduced pulmonary function at 3 months
Source: World J Emerg Surg. 2025 Dec 11;21:5. doi: 10.1186/s13017-025-00667-7 (PMC12801546; doi:10.1186/s13017-025-00667-7)
Supplement: Supplementary file 1 — Supplementary Material 1 [file 13017_2025_667_MOESM1_ESM.docx]

**Supplementary table 1. Factors affecting lung function 3 months post-trauma in patients with and without rib fixation patients**

|  | FVC ≥ 80% | FVC < 80% | p-value |
| --- | --- | --- | --- |
| **Patient with rib fixation (n=73)** | (n=29) | (n=44) |  |
| Patients with flail chest | 13 (44.8%) | 25 (56.8%) | 0.445 |
| Patients with bilateral ribs fracture | 2 (6.9%) | 8 (18.2%) | 0.306 |
| Fracture site |  |  | 0.132 |
| Lateral | 4 (13.8%) | 1 (2.3%) |  |
| Posterior | 5 (17.2%) | 6 (13.6%) |  |
| Combine | 20 (69.0%) | 37 (84.1%) |  |
| Number of fractured ribs | 6.71 ± 2.07 | 7.70 ± 3.03 | 0.134 |
| Patients with severely displaced fractures | 25 (86.2%) | 42 (95.5%) | 0.331 |
| Number of severely displaced fractures | 3.21 ± 2.24 | 4.52 ± 2.49 | 0.025 |
| Lung contusion | 25 (86.2%) | 41 (93.2%) | 0.559 |
| **Patient without rib fixation (n=38)** | (n=20) | (n=18) |  |
| Patients with flail chest | 3 (15.0%) | 5 (27.8%) | 0.571 |
| Patients with bilateral ribs fracture | 1 (5.0%) | 4 (22.2%) | 0.277 |
| Fracture site |  |  | 0.106 |
| Lateral | 9 (45.0%) | 5 (27.8%) |  |
| Posterior | 4 (20.0%) | 1 (5.6%) |  |
| Combine | 7 (35.0%) | 12 (66.7%) |  |
| Number of fractured ribs | 6.35 ± 2.37 | 7.06 ± 2.94 | 0.419 |
| Patients with severely displaced fractures | 13 (65.0%) | 13 (72.2%) | 0.898 |
| Number of severely displaced fractures | 1.45 ± 1.54 | 2.00 ± 1.97 | 0.341 |
| Lung contusion | 10 (50.0%) | 12 (66.7%) | 0.478 |
