# Supplementary material for: Severely displaced rib fractures are independently associated with reduced pulmonary function at 3 months
Source: World J Emerg Surg. 2025 Dec 11;21:5. doi: 10.1186/s13017-025-00667-7 (PMC12801546; doi:10.1186/s13017-025-00667-7)
Supplement: Supplementary file 2 — Supplementary Material 2 [file 13017_2025_667_MOESM2_ESM.docx]

**Supplementary table 2. The Impact of Multiple Severely Displaced Rib Fractures (≥3) on Pulmonary Function 3 Months After Trauma: A Subgroup Analysis of Patients with and without Rib Fixation**

|  | Number of Severely Displaced Rib Fractures < 3 | Number of Severely Displaced Rib Fractures ≥ 3 | p-value |
| --- | --- | --- | --- |
| **Patient with rib fixation (n=73)** | (n=18) | (n=55) |  |
| FVC, (%) | 83.50 ± 21.61 | 72.35 ± 18.64 | 0.038 |
| FVC < 80%, n(%) | 6 (33.3%) | 38 (69.1%) | 0.016 |
| **Patient without rib fixation (n=38)** | (n=29) | (n=9) |  |
| FVC, (%) | 82.31 ± 20.08 | 77.22 ± 23.14 | 0.525 |
| FVC < 80%, n(%) | 13 (44.8%) | 5 (55.6%) | 0.856 |
